# A Novel De Novo *NFKBIA* Missense Mutation Associated to Ectodermal Dysplasia with Dysgammaglobulinemia

**DOI:** 10.3390/genes13101900

**Published:** 2022-10-19

**Authors:** Chai Teng Chear, Bader Abdul Kader El Farran, Marina Sham, Kavetha Ramalingam, Lokman Mohd Noh, Intan Hakimah Ismail, Mei Yee Chiow, Mohd Farid Baharin, Adiratna Mat Ripen, Saharuddin Bin Mohamad

**Affiliations:** 1 Primary Immunodeficiency Unit, Allergy and Immunology Research Centre, Institute for Medical Research, National Institutes of Health, Ministry of Health Malaysia, Setia Alam 40170, Malaysia; 2 Institute of Biological Sciences, Faculty of Science, Universiti Malaya, Kuala Lumpur 50603, Malaysia; 3 Pediatric Department, Kuala Lumpur Hospital, Ministry of Health Malaysia, Kuala Lumpur 50586, Malaysia; 4 Pediatric Department, Taiping Hospital, Ministry of Health Malaysia, Taiping 34000, Malaysia; 5 Department of Paediatrics, Faculty of Medicine and Health Sciences, Universiti Putra Malaysia, Serdang 43400, Malaysia; 6 Centre of Research in Systems Biology, Structural Bioinformatics and Human Digital Imaging (CRYSTAL), Universiti Malaya, Kuala Lumpur 50603, Malaysia

**Keywords:** hyper IgM-like phenotype, *NFKBIA*, IκBα, NF-κB, post-translational modification

## Abstract

Background: Inborn errors of immunity (IEIs) are comprised of heterogeneous groups of genetic disorders affecting immune function. In this report, a 17-month-old Malay patient suspected of having Hyper IgM syndrome, a type of IEIs, was described. However, the diagnosis of Hyper IgM syndrome was excluded by the normal functional studies and the mild features of ectodermal dysplasia observed from a further clinical phenotype inspection. Methods: Whole-exome sequencing (WES) was performed to unravel the causative mutation in this patient. Results: The variant analysis demonstrated a novel missense mutation in *NFKBIA* (NM_020529:c.94A > T,NP_065390:p.Ser32Cys) and was predicted as damaging by in silico prediction tools. The *NFKBIA* gene encodes for IκBα, a member of nuclear factor kappa B (NF-κB) inhibitors, playing an important role in regulating NF-κB activity. The mutation occurred at the six degrons (Asp31-Ser36) in IκBα which were evolutionarily conserved across several species. Prediction analysis suggested that the substitution of Ser32Cys may cause a loss of the phosphorylation site at residue 32 and a gain of the sumoylation site at residue 38, resulting in the alteration of post-translational modifications of IκBα required for NF-κB activation. Conclusion: Our analysis hints that the post-translational modification in the *NFKBIA* Ser32Cys mutant would alter the signaling pathway of NF-κB. Our findings support the usefulness of WES in diagnosing IEIs and suggest the role of post-translational modification of IκBα.

## 1. Introduction

Inborn errors of immunity (IEIs), previously known as primary immunodeficiencies (PIDs), represent a class of genetic disorders of immune function rendering patients susceptible to various infections, autoimmunity, allergies, and/or cancer [1]. According to the International Union of Immunological Societies (IUIS), a total of 485 IEIs have been reported arising from defects in more than 490 genes [2]. The use of next-generation sequencing (NGS), particularly whole-exome sequencing (WES), has improved clinical diagnosis and unraveled novel genes and variants underlying IEIs [3,4,5].

Hypohidrotic ectodermal dysplasia (HED) (OMIM:305100) is a genetic disorder characterized by variable features of ectodermal dysplasia such as sparse hair, nail and tooth anomalies, decreased skin pigment, and reduced number of sweat glands. This phenotype can be caused by mutations in the genes involved in two signaling pathways (i.e., ectodysplasin (EDA) pathways and nuclear factor kappa B essential modulator (NEMO) regulatory pathway) that modulate nuclear factor kappa B (NF-κB) activity or changes in the transcription and/or expression regulators of the genes involved in the ectodermal development or changes in the function of structural protein in the cell membrane. Mutations in these 16 genes (Table S1) have been reported to impede the normal development of ectodermal-derived structures [6,7], while mutations in three of these genes (i.e., *NEMO/ inhibitor of nuclear factor kappa B kinase regulatory subunit γ (IKBKG)*, *nuclear factor kappa B inhibitor α (NFKBIA),* and *inhibitor of nuclear factor kappa B kinase subunit β (IKBKB)*) result in anhidrotic ectodermodysplasia with immunodeficiency (EDA-ID) [2]. EDA-ID (OMIM: 612132) is a genetic disorder characterized by variable features of ectodermal dysplasia such as sparse hair, tooth anomalies, decreased skin pigment, a reduced number of sweat glands, and varied immunologic and infectious manifestations with different severity [8]. Mutation in *NEMO* is associated with X-linked EDA-ID, while mutations in *NFKBIA* and *IKBKB* were related to autosomal dominant EDA-ID [2]. Mutations in any of these three genes may affect the NF-κB activity that regulates the expression of genes which modulate immunity, inflammatory response, proliferation, and apoptosis [9].

In addition to having ectodermal structural defects, some patients with *NEMO* or *NFKBIA* mutation can present with a Hyper-IgM-type phenotype [2]. In resting cells, the NF-κB activation is prohibited by the NF-κB inhibitory protein (IκB). Following the interaction of the CD40 ligand and CD40, the NEMO-encoded IκB kinase (IKK) phosphorylates IκB, leading to the degradation of IκB. Then, the NF-κB is translocated to the nucleus to induce the expression of the target genes, such as *AID* and *UNG,* resulting in immunoglobulin class switching and somatic hypermutation. In the presence of *NEMO* or *NFKBIA* mutation, defective NF-κB nuclear translocation may hamper the downstream immunoglobulin class switch recombination and somatic hypermutation [10].

Thus far, thirteen distinct mutations in *NFKBIA* have been associated with EDA-ID [11,12,13]. To the best of our knowledge, this is the first report from Southeast Asia describing the clinical and immunological findings of a Malaysian boy caused by *NFKBIA* mutation.

## 2. Materials and Methods

### 2.1. Patient

This study was approved by the Medical Research and Ethics Committee (MREC), Malaysia (KKM/NIHSEC/P20-2460). A patient exhibiting hyper-IgM-like phenotypes was enrolled in this study. Peripheral venous blood was withdrawn from the patient and both parents after obtaining informed consent.

### 2.2. Whole-Exome Sequencing and Bioinformatics Analysis

Genomic DNA was isolated from the patient’s peripheral blood mononuclear cells (PBMC) using the QIAamp DNA Blood Mini Kit (Qiagen, Germany) according to the manufacturer’s protocol. The sequencing library was prepared using the Agilent SureSelect Human All Exon V5 kit (Agilent Technologies, Santa Clara, CA, USA) and sequenced using the Illumina Hiseq 4000 sequencer (Illumina, San Diego, CA, USA). Raw reads generated were aligned to the human reference genome GRCh38 using BWA [14]. Post-alignment processes, such as mark duplication and base quality score recalibration, were performed with Picard (http://broadinstitute.github.io/picard, accessed on 11 November 2019) and GATK 4.0 [15] respectively. Then, all variants such as single-nucleotide variants (SNVs) and small insertions–deletions (indels) were called using GATK HaplotypeCaller. Variant annotation was performed with wANNOVAR [16]. Variant prioritization was performed using the filtering strategy described previously [17]. In addition, combined annotation-dependent depletion (CADD) was used to evaluate the variant deleteriousness [18].

### 2.3. Variant Validation via Sanger Sequencing

The *NFKBIA* gene (NG_007571.1) was amplified from the patient’s and parents’ genomic DNA by PCR using the following primers: forward primer (5′- CGCCCCAGCGAGGAAGCAG-3′) and reverse primer (5′- CCTCCGCCACTTACGAGTC-3′) with an annealing temperature of 61 °C. The PCR product was then sequenced with bidirectional Sanger sequencing.

### 2.4. Conservation Analysis

To identify a conserved region in the IκBα protein across species, evolutionary conservation analysis was performed using Clustal Omega version 1.2.4 [19].

### 2.5. Structural Effect Evaluation of Ser32 Variants

Project HOPE (https://www3.cmbi.umcn.nl/hope/, accessed on 1 August 2022) is a webserver that provides the protein annotation and structural information based on the UniProt database and Alphafold2 prediction [20]. The full-length amino acid sequence of IkBα (UniProt ID: P25963) was used as the input. The protein structure, structural consequence of the mutant variant (Ser32Cys), and the previously reported variants at the same mutation site (i.e., Ser32Ile, Ser32Gly, Ser32Asn, and Ser32Arg) were analyzed, respectively.

### 2.6. Phosphorylation Prediction

To predict the impact of single-nucleotide variation on the phosphorylation site, the identified *NFKBIA* variant in this study (Ser32Cys) was analyzed using mutation impact on phosphorylation (MIMP) (http://mimp.baderlab.org/, accessed on 13 October 2021). MIMP is a web server that predicts if a mutation within a phosphorylation site will affect which kinase binds to that site, as well as the impact of the mutation on phosphorylation [21].

### 2.7. Post-Translational Modification Site Prediction

To further investigate if the Ser32Cys mutant affects other types of post-translational modification of IκBα, the sites involved in protein post-translational modification were predicted using the MusiteDeep server (https://www.musite.net/, accessed on 9 December 2021). The MusiteDeep server uses a deep-learning framework to perform knowledge-based prediction and visualization of protein post-translational modification (PTM) sites such as phosphorylation, glycosylation, ubiquitination, sumoylation, acetylation, methylation, pyrrolidone carboxylic acid, palmitoylation, and hydroxylation [22,23]. A predicted gain or loss of a PTM site had a joint probability value above the default cut-off (0.5). Predictions of all twelve PTM types were performed in this study.

## 3. Results

### 3.1. Clinical Features

The patient was the only child of healthy non-consanguineous parents. He was born at full-term with a birth weight of 3.35 kg. He developed respiratory distress soon after delivery, which was treated as congenital pneumonia, requiring low-flow oxygen therapy until one month of age. He had persistent oral and perianal thrush. Conjunctival scraping was positive for chlamydia. There were no sequelae after receiving bacille Calmette–Guérin (BCG) vaccination. He presented again at five months old with severe respiratory distress, requiring non-invasive respiratory support. Chest radiography revealed severe pneumonia with bilateral pleural effusion. He was hospitalized for three months due to septicemia caused by *Staphylococcus aureus* and *Klebsiella pneumoniae*; melioidosis with multiple microabscesses in the liver, spleen, and kidneys; as well as possible pulmonary aspergillosis. Toxoplasma, rubella, cytomegalovirus, and herpes simplex virus (TORCH) screening was negative. Echocardiography showed pericardial effusion with cardiomegaly, which subsided without intervention. He was further investigated at this point when a routine immunological workup showed an elevated IgM level with low IgG and IgA levels, prompting the need to consider hyper IgM syndrome (Table 1). He was later started on intravenous immunoglobulin replacement therapy (0.5 mg/kg every 3 weeks) along with prophylactic antibiotic co-trimoxazole and antifungal itraconazole. However, the flow cytometric analysis demonstrated the presence of CD40 ligand expression on activated CD4+ T cells (Figure S1) and CD40 protein expression on B cells (Figure S2), excluding the diagnosis of hyper IgM syndrome. At 11 months old, he was admitted again due to septic shock, and a blood culture grew *Enterobacter gergoviae*. Two months after hospital discharge, he was found to have hypothyroidism and treated with L-thyroxine. The patient was admitted again to the local hospital at 17 months old due to bronchopneumonia. In view of the recurrent, persistent, and severe infections, he was referred to a tertiary hospital. This time, he was discovered to have sparse hair, thin eyebrows, low-set ears, dry skin, and absent dentition. However, no abnormality was observed in his fingers and nails. His condition continued to deteriorate, and he developed worsening respiratory distress with heart failure. Later, he developed autoimmune hemolytic anemia and thrombocytopenia, and blood cultures persistently grew *Salmonella sp.* Subsequently, he succumbed to multidrug-resistant *Enterobacter sp.* and *K. pneumoniae* septicemia after 19 days of admission.

### 3.2. Bioinformatics Analysis

**Figure 1:**
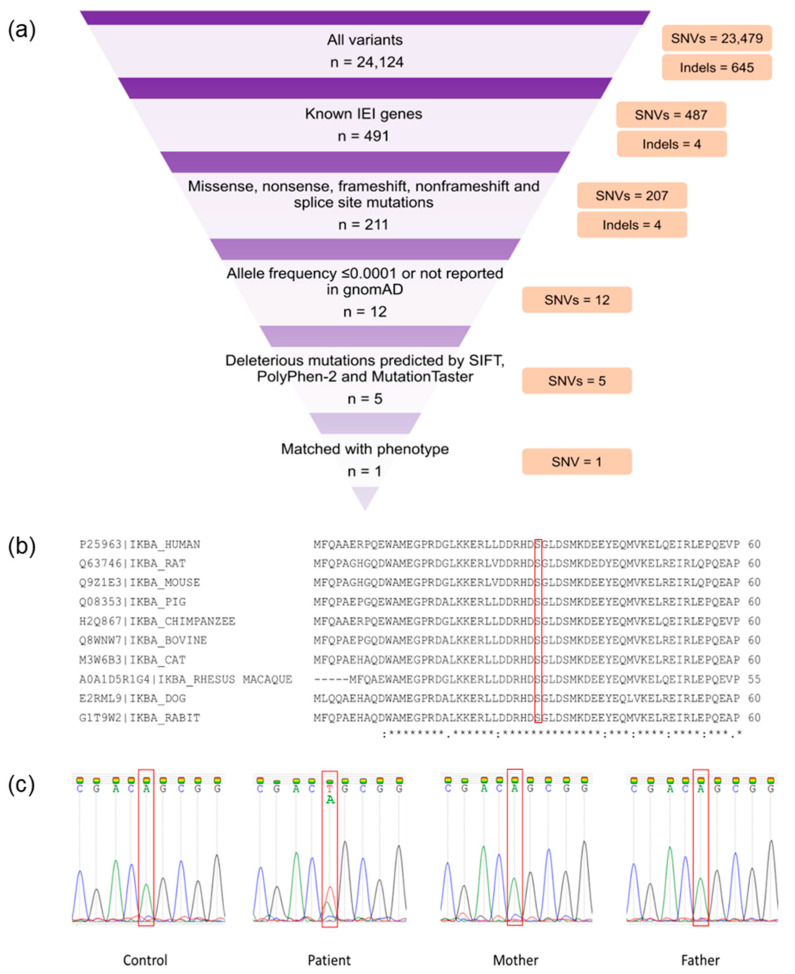
Whole-exome sequencing (WES) findings and variant validation. (**a**) WES filtering diagram. (**b**) Conservation analysis of NF-κB inhibitor α (IκBα). The red bracket indicates the mutation site (p.Ser32). A fully conserved residue is indicated by an asterisk sign (*). A colon sign (:) indicates conservation between groups of strongly similar properties, while a period sign (.) indicates conservation between groups of weakly similar properties. (**c**) Familial segregation analysis by Sanger sequencing. The red bracket shows the mutation site (c.94A). The patient had a de novo heterozygous Ser32Cys (c.94A > T) mutation.

Whole-exome sequencing generated 66,450,938 paired-end reads; 99.09% (*n* = 65,844,546) were properly paired and mapped to the human reference genome. A total of 24,008 exonic variants were detected in the patient, including 23,401 SNVs and 607 indels. Additionally, the patient had 116 splicing variants encompassing 78 SNVs and 38 indels. The candidate variants (Table S2) were prioritized from overall SNVs and indels based on the criteria as described previously [17]. A heterozygous missense mutation in *NFKBIA* (NM_020529.3:exon 1:c.94A > T:p.Ser32Cys) that matched with the patient’s phenotype was identified (Figure 1a). This mutation was predicted to be damaging by SIFT, PolyPhen-2, and MutationTaster. In addition, the deleteriousness of this variant was indicated by a high Phred-scaled CADD score (i.e., 26.1). The c.94A > T *NFKBIA* variant was not reported in the gnomAD. The mutation site was also conserved among several species (Figure 1b). Familial segregation analysis revealed the absence of mutant alleles in both parents, inferring a de novo *NFKBIA* mutation in the patient (Figure 1c). According to the American College of Medical Genetics and Genomics (ACMG) guideline, this novel variant was classified as pathogenic.

### 3.3. Structural Effect Evaluation of Ser32 Variants

**Figure 2:**
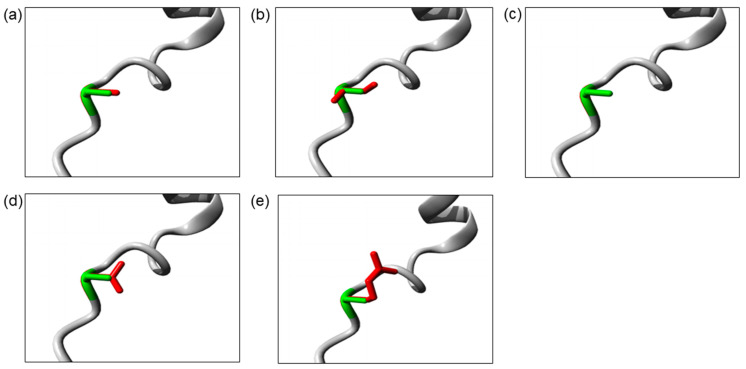
The close-up image of superimposed structure of wild-type and mutant residues. (**a**) Ser32Cys. (**b**) Ser32Ile. (**c**) Ser32Gly. (**d**) Ser32Asn. (**e**) Ser32Arg. The protein core is shown in gray while the amino acid side chain of the wild-type (green) and the mutant (red) residue are represented as sticks.

The Ser32 residue is located at the destructive motif that is important for ubiquitin-dependent IκBα degradation. The Ser32Cys and Ser32Ile mutations cause the change in serine into a more hydrophobic residue at position 32. The isoleucine residue is bigger than the wild-type serine residue, which may disturb its molecular interactions, whereas the Ser32Gly mutation introduces a smaller glycine residue that is very flexible. Hence, it may affect the requisite rigidity of the IκBα protein, causing a possible loss of external interactions. On the other hand, the Ser32Asn and Ser32Arg mutations introduce a bigger and less hydrophobic residue at position 32. This change may affect the interactions with other molecules or other parts of the protein. The change in hydrophobicity may affect the function of the molecule through loss of hydrophobic interactions. In addition, the Ser32Arg mutation results in the change in a neutral serine to a positively charged arginine. The difference in polarity may create repulsion between the mutant residue and the neighboring residues. The three-dimensional models of these IκBα mutant proteins are illustrated in Figure 2.

### 3.4. Post-Translational Modification Site Predictions

**Figure 3:**
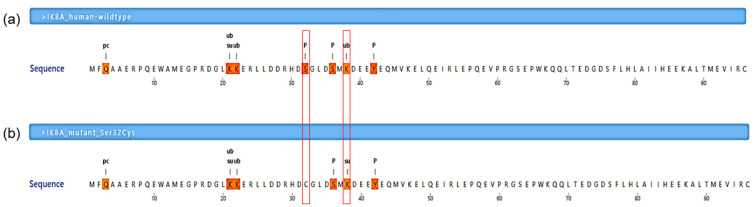
Post-translational modification prediction by a deep-learning algorithm. (**a**) A segment of human NF-κB inhibitor α (IκBα) wild-type protein sequence (residues Met1-Cys96 are shown). (**b**) A segment of human IκBα mutant protein (Ser32Cys) sequence (residues Met1-Cys96 are shown). The symbol pc denotes the pyrrolidone carboxylic acid site, ub denotes the ubiquitination site, su denotes the sumoylation site, and p denotes the phosphorylation site. The mutation was predicted to abolish the phosphorylation site at residue 32 and gain a sumoylation site at residue 38 (these two residues are indicated in the red line boxes).

The Ser32Cys mutation was predicted to abolish the existing phosphorylation site at residue 32 for 25 kinases, including the IκB kinase (IKK) complex, as shown by MIMP server (Table 2). Furthermore, it was consistent with the prediction from MusiteDeep server, which also showed that the Ser32Cys mutant had a loss of phosphorylation site at residue 32 (Figure 3). In addition to that, the wild-type IκBα had a ubiquitination site at lysine-38 residue (PTM score: 0.513), while the Ser32Cys mutant lost its ubiquitination site and introduced a sumoylation site at lysine-38 residue (PTM score: 0.555).

## 4. Discussion

In this study, we report a 17-month-old Malay boy who presented with dysgammaglobulinemia and recurrent fungal and bacterial infections. In addition to that, the patient exhibited less apparent features of ectodermal dysplasia (ED), such as sparse hair and eyebrows, dry skin, and an absence of teeth, as observed at the age of 17 months old. ED is a rare inherited disorder characterized by abnormalities in tissues derived from the ectoderm, such as teeth, skin, hair, nail, and sweat glands. The estimated incidence of ED was 7 in 10,000 births [6]. The clinical manifestations may vary from mild to severe forms [24]. Sixteen genes (*ED-1, EDAR, EDARADD, NEMO/IKBKG, NFKBIA, IKBKB, p63, DLX3, MSX1, EVC2, EVC, GJB6, PVRL1, PKP1, CDH3*, and *WNT10A*) (Table S1) have been described to cause ED [6,7]. Whole-exome sequencing revealed a novel pathogenic variant in *NFKBIA* related to ED in the patient (Table S3). Segregation analysis confirmed that the identified variant was a de novo mutation, explaining the absence of family history for ED.

Nuclear factor kappa B (NF-κB) is constituted by a group of dimeric transcription factors that regulates the expression of genes essential for immune and inflammatory response, developmental processes, cellular growth, and apoptosis. Depending on the types of stimuli, NF-κB activation involves two major signaling pathways, the canonical and non-canonical pathways. Activation of the canonical NF-κB signaling pathway is tightly regulated by the inhibitor protein of NF-κB, such as NF-κB inhibitor α (IκBα) [25]. IκBα, which belongs to the serine/theorine protein kinase family, is encoded by the *NFKBIA* gene. The IκBα protein contains an N-terminal signal–receiving domain, followed by an ankyrin repeat domain, a C-terminal proline–glutamic–serine–threonine (PEST) sequence, and two nuclear export sequences [26,27,28]. In unstimulated cells, the NF-κB dimer is attached with IκBα, forming an inactive trimer complex in the cytoplasm. External stimuli, such as microbial components, mitogens, cytokines, stress agents, and growth factors, can activate the canonical NF-κB pathway [29]. The phosphorylation of IκBα by IKK induces the ubiquitin-dependent degradation of IκBα by proteasome, leading to the rapid and transient release and nuclear translocation of active NF-κB dimers where it drives the transcription of a target gene that mediates an immune or inflammatory response [30].

The IκBα gain-of-function mutation was first described in 2003 [31]. To date, twenty patients with *NFKBIA* mutations have been reported in the literature [11,12,13,32]. Most of the patients have de novo mutation in *NFKBIA*, except for three patients who inherited the mutant allele from their father [11,32]. Most patients with *NFKBIA* mutations manifested anhidrotic ectodermal dysplasia (EDA) features and had high susceptibility to fungal, pyogenic, mycobacterial, and viral infections, as early as three months old, due to immunodeficiency [11]. Some patients may exhibit hyper-IgM phenotypes due to defective NF-kB-mediated signaling pathway [31,33,34,35,36]. The mutation involving codon 32 of *NFKBIA* is the most frequent mutation, reported in approximately 35% of patients with gain-of-function *NFKBIA* mutation. The patients with Ser32Ile [31,35], Ser32Gly [37], Ser32Arg, and Ser32Asn [38] mutation in *NFKBIA* manifested features of EDA with immunodeficiency. Two patients with Ser32Ile mutation had high IgM levels, similar to our patient, exhibiting Hyper IgM-like immunodeficiency syndrome [11]. Furthermore, point mutations at or adjacent to Ser32 and Ser36 appeared to have more severe phenotypes and poor clinical outcomes after hematopoietic stem cell transplantation [39]. Our patient succumbed to sepsis before the age of two.

**Figure 4:**
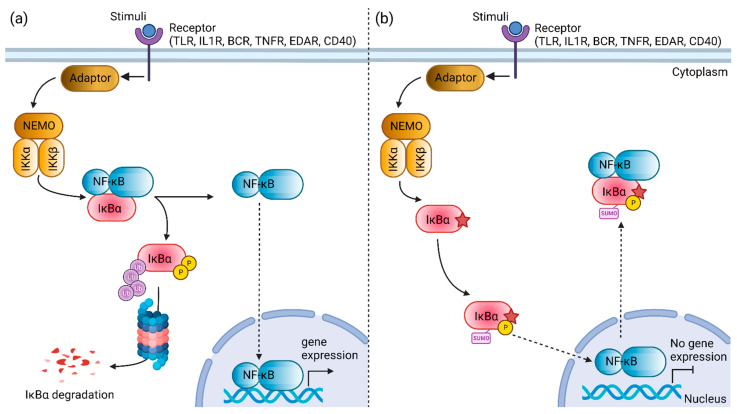
Schematic diagram illustrating the nuclear factor kappa B (NF-κB) signaling pathway mediated by the NF-κB inhibitor α (IκBα) protein. (**a**) The constitutive NF-κB activation is mediated by the degradation of the IκBα protein. (**b**) The mutant Ser32Cys IκBα protein (star symbol) is speculated to exhibit changes in the post-translational modifications. The loss of phosphorylation site at residue 32 and introduction of a new sumoylation site at residue 38 may facilitate the nuclear transport of NF-κB to the cytoplasm, resulting in the inhibition of NF-κB activation. TLR, toll-like receptor; IL1R, interleukin-1 receptor; BCR, B cell receptor; TNFR, tumor necrosis factor receptor; EDAR, ectodysplasin A receptor; NEMO, NF-κB essential modulator; IKKα, IkappaB kinase α subunit; IKKβ, IkappaB kinase β subunit; NF-κB, nuclear factor kappa B; IκBα, NF-κB inhibitor α. Created with BioRender.com, accessed on 30 July 2022.

Evolutionary conservational analysis showed that the six-amino-acid degron (31st–36th residues, DSGLDS) in the N-terminal of IκBα is evolutionarily conserved (Figure 1b). Of these six amino acid residues, residues Ser32 and Ser36 are important phosphor-acceptor sites in IκBα [28,40]. Once phosphorylated, the phosphorylation-based motif (DpSGXXpS) directs the recognition of the E3 complex that facilitated it to be ubiquitinated and marked it for proteasomal degradation [41]. HOPE analysis demonstrated that the mutations (Ser32Cys, Ser32Ile, Ser32Gly, Ser32Asn, and Ser32Arg) introduce an amino acid with different characteristics, such as hydrophobicity, size, and/or polarity, which may disrupt the function of the IκBα protein. Increased IκBα and reduced phospho-IκBα following tumor necrosis factor α (TNFα) simulation in vitro were observed in the patients with Ser32Ile [37], Ser32Arg, and Ser32Asn mutation [38]. In addition, these patients had significant inhibitory effects on NF-κB signaling [38]. The loss of phosphorylation site at residue 32 hindered the phosphorylation by the IKK complex, as predicted by the MIMP server. A previous study showed that phosphorylation of Ser32 and Ser36 residues was required prior to the ubiquitination-dependent degradation of IκBα [40]. Deep-learning-based protein post-translational modification site prediction using the MuSiteDeep server showed that the Ser32Cys mutant had losses of phosphorylation site at residue 32 and ubiquitination site at residue 38. It is speculated that the downstream ubiquitin-dependent proteasomal degradation might be affected, hindering the liberation of NF-κB dimers for nuclear translocation. On the other hand, the Ser32Cys mutant was predicted to gain a sumoylation site at residue 38 (Figure 4). Sumoylation is a post-translational modification that adds small ubiquitin-related modifiers (SUMOs) to lysine residues in the target proteins, regulating the protein stability, solubility, nuclear–cytosolic localization, and protein-binding activity [42]. Sumoylation may be involved in the transfer of IκBα to the nucleus, where it binds to NF-κB complexes, inactivating them and shuttling them to the cytoplasm [43]. A previous study showed that sumoylation was able to inhibit IκB degradation by impairing transcriptional activation of nitric oxide synthase II synthesis by NF-κB [44]. Nitric oxide is essential for effective innate immunity against fungi, *Klebsiella*, and *Salmonella* [45,46,47]. Therefore, recurrent fungal and bacterial infections seen in our patient may be postulated by changes in the post-translational modification of IκB.

## 5. Conclusions

In conclusion, whole-exome sequencing identified a novel *NFKBIA* Ser32Cys mutation in a patient exhibiting mild features of ED with immunodeficiency. Milder forms of ED may be unrecognized during early infancy; an early molecular diagnosis can direct timely multidisciplinary management. Despite having mild ED features, the patient’s condition deteriorated, and he died at a young age due to sepsis. This is consistent with previous studies where patients with Ser32 mutation in *NFKBIA* tend to develop severe clinical phenotypes. It is speculated that the Ser32 point mutation leads to the changes in the post-translational modification of IκBα, hence disrupting NF-κB activation. Therefore, the role of post-translational modification in IκBα degradation warrants further investigation in future studies.

## Figures and Tables

**Table 1:** Immunological parameters of the study subject.

Immunological Parameters	Result	Reference Range
Lymphocyte subsets (Absolute count, ×10^6^ cells/L)		
Total T cells	11,529	1700–3600
Total B cells	1099	500–1500
CD4 cells	9555	1700–2800
CD8 cells	3647	800–1200
NK cells	223	300–700
Immunoglobulins		
IgG (g/L)	0.91	2.6–15.2
IgA (g/L)	0.08	0.16–1.1
IgM (g/L)	5.85	0.10–1.2
IgE (kU/L)	4.43	<7.3
Complements (g/L)		
C3	1.55	0.5–0.9
C4	0.29	0.1–0.4

**Table 2:** The impact of Ser32Cys mutation on kinase–substrate interactions by the mutations impact on phosphorylation (MIMP) server.

Gene	Mutation	Phosphorylation Site	Name of Kinase	Family of Kinase	Joint Probability *	Effect
*NFKBIA*	Ser32Cys	32	CHUK	IKK	0.9997	loss
*NFKBIA*	Ser32Cys	32	TGFBR2	STKR_Type2	0.9829	loss
*NFKBIA*	Ser32Cys	32	IKBKE	IKK	0.9809	loss
*NFKBIA*	Ser32Cys	32	PRKACA	PKA	0.9375	loss
*NFKBIA*	Ser32Cys	32	IKBKB	IKK	0.9338	loss
*NFKBIA*	Ser32Cys	32	PRKAA1	CAMKL_AMPK	0.8779	loss
*NFKBIA*	Ser32Cys	32	CSNK2A1	CK2	0.8514	loss
*NFKBIA*	Ser32Cys	32	DAPK3	DAPK_DAPK	0.8496	loss
*NFKBIA*	Ser32Cys	32	PRKCE	PKC_Eta	0.8392	loss
*NFKBIA*	Ser32Cys	32	CAMK1	CAMK1	0.8206	loss
*NFKBIA*	Ser32Cys	32	PRKD1	PKD	0.8176	loss
*NFKBIA*	Ser32Cys	32	RPS6KB1	RSK_p70	0.8101	loss
*NFKBIA*	Ser32Cys	32	MAP3K8	STE-Unique	0.8001	loss
*NFKBIA*	Ser32Cys	32	CAMK2A	CAMK2	0.7852	loss
*NFKBIA*	Ser32Cys	32	CHEK1	CAMKL_CHK1	0.7832	loss
*NFKBIA*	Ser32Cys	32	CAMK4	CAMK1	0.7608	loss
*NFKBIA*	Ser32Cys	32	SGK1	SGK	0.7565	loss
*NFKBIA*	Ser32Cys	32	PRKAA2	CAMKL_AMPK	0.6896	loss
*NFKBIA*	Ser32Cys	32	AKT2	Akt	0.6866	loss
*NFKBIA*	Ser32Cys	32	PAK2	STE20_PAKA	0.6696	loss
*NFKBIA*	Ser32Cys	32	RPS6KA3	RSK_RSK	0.6637	loss
*NFKBIA*	Ser32Cys	32	PRKCD	PKC_Delta	0.6406	loss
*NFKBIA*	Ser32Cys	32	PIM1	PIM	0.5352	loss
*NFKBIA*	Ser32Cys	32	CSNK2A2	CK2	0.5298	loss
*NFKBIA*	Ser32Cys	32	PAK1	STE20_PAKA	0.5292	loss

* Phosphorylation loss is predicted if the joint probability *p* > 0.5.

## Data Availability

The data that support the findings of this study are available in online repository (https://www.ncbi.nlm.nih.gov/, assessed on 10 June 2022, PRJNA847876).

## Supplementary Materials

The following supporting information can be downloaded at: https://www.mdpi.com/article/10.3390/genes13101900/s1. Figure S1: Cell surface expression of CD154 (CD40 ligand) on activated CD3+ CD4+ T-cells from healthy control and patient. Figure S2: Flow cytometric analysis of CD40 expression in healthy control and patient. Table S1: Genes responsible for Ectodermal Dysplasias. Table S2: List of five rare variants predicted to be damaging using in silico tools. Table S3: List of variants in the genes related to ectodermal dysplasia.

## References

[1] Bousfiha A., Jeddane L., Picard C., Al-Herz W., Ailal F., Chatila T., Cunningham-Rundles C., Etzioni A., Franco J.L., Holland S.M. (2020). Human Inborn Errors of Immunity: 2019 Update of the IUIS Phenotypical Classification. J. Clin. Immunol..

[2] Tangye S.G., Al-Herz W., Bousfiha A., Cunningham-Rundles C., Franco J.L., Holland S.M., Klein C., Morio T., Oksenhendler E., Picard C. (2022). Human Inborn Errors of Immunity: 2022 Update on the Classification from the International Union of Immunological Societies Expert Committee. J. Clin. Immunol..

[3] Engelbrecht C., Urban M., Schoeman M., Paarwater B., van Coller A., Abraham D.R., Cornelissen H., Glashoff R., Esser M., Möller M. (2021). Clinical Utility of Whole Exome Sequencing and Targeted Panels for the Identification of Inborn Errors of Immunity in a Resource-Constrained Setting. Front. Immunol..

[4] Meyts I., Bosch B., Bolze A., Boisson B., Itan Y., Belkadi A., Pedergnana V., Moens L., Picard C., Cobat A. (2016). Exome and Genome Sequencing for Inborn Errors of Immunity. J. Allergy Clin. Immunol..

[5] Ripen A.M., Chear C.T., Baharin M.F., Nallusamy R., Chan K.C., Kassim A., Choo C.M., Wong K.J., Fong S.M., Tan K.K. (2021). A Single-center Pilot Study in Malaysia on the Clinical Utility of Whole-exome Sequencing for Inborn Errors of Immunity. Clin. Exp. Immunol..

[6] García-Martín P., Hernández-Martín A., Torrelo A. (2013). Ectodermal Dysplasias: A Clinical and Molecular Review. Actas Dermo-Sifiliográficas.

[7] Cardinez C., Miraghazadeh B., Tanita K., da Silva E., Hoshino A., Okada S., Chand R., Asano T., Tsumura M., Yoshida K. (2018). Gain-of-Function IKBKB Mutation Causes Human Combined Immune Deficiency. J. Exp. Med..

[8] Kawai T., Nishikomori R., Heike T. (2012). Diagnosis and Treatment in Anhidrotic Ectodermal Dysplasia with Immunodeficiency. Allergol. Int..

[9] Zinatizadeh M.R., Schock B., Chalbatani G.M., Zarandi P.K., Jalali S.A., Miri S.R. (2021). The Nuclear Factor Kappa B (NF-KB) Signaling in Cancer Development and Immune Diseases. Genes Dis..

[10] Etzioni A., Ochs H.D. (2004). The Hyper IgM Syndrome—An Evolving Story. Pediatr. Res..

[11] Boisson B., Puel A., Picard C., Casanova J.-L. (2017). Human IκBα Gain of Function: A Severe and Syndromic Immunodeficiency. J. Clin. Immunol..

[12] Batlle-Masó L. (2020). Genetic Diagnosis of Autoinflammatory Disease Patients Using Clinical Exome Sequencing. Eur. J. Med. Genet..

[13] Wen W., Wang L., Deng M., Li Y., Tang X., Mao H., Zhao X. (2022). A Heterozygous N-Terminal Truncation Mutation of NFKBIA Results in an Impaired NF-ΚB Dependent Inflammatory Response. Genes Dis..

[14] Li H., Durbin R. (2009). Fast and Accurate Short Read Alignment with Burrows-Wheeler Transform. Bioinformatics.

[15] McKenna A., Hanna M., Banks E., Sivachenko A., Cibulskis K., Kernytsky A., Garimella K., Altshuler D., Gabriel S., Daly M. (2010). The Genome Analysis Toolkit: A MapReduce Framework for Analyzing next-Generation DNA Sequencing Data. Genome Res..

[16] Chang X., Wang K. (2012). WANNOVAR: Annotating Genetic Variants for Personal Genomes via the Web. J. Med. Genet..

[17] Chear C.T., Nallusamy R., Canna S.W., Chan K.C., Baharin M.F., Hishamshah M., Ghani H., Ripen A.M., Mohamad S.B. (2020). A Novel de Novo NLRC4 Mutation Reinforces the Likely Pathogenicity of Specific LRR Domain Mutation. Clin. Immunol..

[18] Rentzsch P., Witten D., Cooper G.M., Shendure J., Kircher M. (2019). CADD: Predicting the Deleteriousness of Variants throughout the Human Genome. Nucleic Acids Res..

[19] Sievers F., Wilm A., Dineen D., Gibson T.J., Karplus K., Li W., Lopez R., McWilliam H., Söding J., Thompson J.D. (2011). Fast, Scalable Generation of High-Quality Protein Multiple Sequence Alignments Using Clustal Omega. Mol. Syst. Biol..

[20] Venselaar H., te Beek T.A., Kuipers R.K., Hekkelman M.L., Vriend G. (2010). Protein Structure Analysis of Mutations Causing Inheritable Diseases. An e-Science Approach with Life Scientist Friendly Interfaces. BMC Bioinform..

[21] Wagih O., Reimand J., Bader G.D. (2015). MIMP: Predicting the Impact of Mutations on Kinase-Substrate Phosphorylation. Nat. Methods.

[22] Wang D., Zeng S., Xu C., Qiu W., Liang Y., Joshi T., Xu D. (2017). MusiteDeep: A Deep-Learning Framework for General and Kinase-Specific Phosphorylation Site Prediction. Bioinformatics.

[23] Wang D., Liu D., Yuchi J., He F., Jiang Y., Cai S., Li J., Xu D. (2020). MusiteDeep: A Deep-Learning Based Webserver for Protein Post-Translational Modification Site Prediction and Visualization. Nucleic Acids Res..

[24] Prashanth S., Deshmukh S. (2012). Ectodermal Dysplasia: A Genetic Review. Int. J. Clin. Pediatr. Dent..

[25] Yu H., Lin L., Zhang Z., Zhang H., Hu H. (2020). Targeting NF-ΚB Pathway for the Therapy of Diseases: Mechanism and Clinical Study. Signal Transduct. Target. Ther..

[26] Truhlar S.M.E., Torpey J.W., Komives E.A. (2006). Regions of IkBa That Are Critical for Its Inhibition of NF-KB.DNA Interaction Fold upon Binding to NF-KB. Proc. Natl. Acad. Sci. USA.

[27] Wang X., Peng H., Huang Y., Kong W., Cui Q., Du J., Jin H. (2020). Post-Translational Modifications of IκBα: The State of the Art. Front. Cell Dev. Biol..

[28] Yazdi S., Naumann M., Stein M. (2017). Double Phosphorylation-Induced Structural Changes in the Signal-Receiving Domain of IκBα in Complex with NF-ΚB: Double-Phosphorylation Events in the SRD of IκBα. Proteins.

[29] Liu T., Zhang L., Joo D., Sun S.-C. (2017). NF-ΚB Signaling in Inflammation. Signal Transduct. Target. Ther..

[30] Kanarek N., Ben-Neriah Y. (2012). Regulation of NF-ΚB by Ubiquitination and Degradation of the IκBs: IκB Ubiquitination and Degradation. Immunol. Rev..

[31] Courtois G., Smahi A., Reichenbach J., Döffinger R., Cancrini C., Bonnet M., Puel A., Chable-Bessia C., Yamaoka S., Feinberg J. (2003). A Hypermorphic IκBα Mutation Is Associated with Autosomal Dominant Anhidrotic Ectodermal Dysplasia and T Cell Immunodeficiency. J. Clin. Investig..

[32] Sogkas G., Adriawan I.R., Ringshausen F.C., Baumann U., Schröder C., Klemann C., von Hardenberg S., Schmidt G., Bernd A., Jablonka A. (2020). A Novel NFKBIA Variant Substituting Serine 36 of IκBα Causes Immunodeficiency with Warts, Bronchiectasis and Juvenile Rheumatoid Arthritis in the Absence of Ectodermal Dysplasia. Clin. Immunol..

[33] Dupuis-Girod S., Cancrini C., Le Deist F., Palma P., Bodemer C., Puel A., Livadiotti S., Picard C., Bossuyt X., Rossi P. (2006). Successful Allogeneic Hemopoietic Stem Cell Transplantation in a Child Who Had Anhidrotic Ectodermal Dysplasia With Immunodeficiency. Pediatrics.

[34] Giancane G., Ferrari S., Carsetti R., Papoff P., Iacobini M., Duse M. (2013). Anhidrotic Ectodermal Dysplasia: A New Mutation. J. Allergy Clin. Immunol..

[35] Janssen R., van Wengen A., Hoeve M.A., ten Dam M., van der Burg M., van Dongen J., van de Vosse E., van Tol M., Bredius R., Ottenhoff T.H. (2004). The Same IκBα Mutation in Two Related Individuals Leads to Completely Different Clinical Syndromes. J. Exp. Med..

[36] Schimke L.F., Rieber N., Rylaarsdam S., Cabral-Marques O., Hubbard N., Puel A., Kallmann L., Sombke S.A., Notheis G., Schwarz H.-P. (2013). A Novel Gain-of-Function IKBA Mutation Underlies Ectodermal Dysplasia with Immunodeficiency and Polyendocrinopathy. J. Clin. Immunol..

[37] Staples E., Morillo-Gutierrez B., Davies J., Petersheim D., Massaad M., Slatter M., Dimou D., Doffinger R., Hackett S., Kumararatne D. (2017). Disseminated Mycobacterium Malmoense and Salmonella Infections Associated with a Novel Variant in NFKBIA. J. Clin. Immunol..

[38] Moriya K., Sasahara Y., Ohnishi H., Kawai T., Kanegane H. (2018). IKBA S32 Mutations Underlie Ectodermal Dysplasia with Immunodeficiency and Severe Noninfectious Systemic Inflammation. J. Clin. Immunol..

[39] Petersheim D., Massaad M.J., Lee S., Scarselli A., Cancrini C., Moriya K., Sasahara Y., Lankester A.C., Dorsey M., Di Giovanni D. (2018). Mechanisms of Genotype-Phenotype Correlation in Autosomal Dominant Anhidrotic Ectodermal Dysplasia with Immune Deficiency. J. Allergy Clin. Immunol..

[40] Chen Z., Hagler J., Palombella V.J., Melandri F., Scherer D., Ballard D., Maniatis T. (1995). Signal-Induced Site-Specific Phosphorylation Targets I Kappa B Alpha to the Ubiquitin-Proteasome Pathway. Genes Dev..

[41] Yaron A., Hatzubai A., Davis M., Lavon I., Amit S., Manning A.M., Andersen J.S., Mann M., Mercurio F., Ben-Neriah Y. (1998). Identifcation of the Receptor Component of the IkBa Ubiquitin Ligase. Nature.

[42] Celen A.B., Sahin U. (2020). Sumoylation on Its 25th Anniversary: Mechanisms, Pathology, and Emerging Concepts. FEBS J..

[43] Perkins N.D. (2006). Post-Translational Modifications Regulating the Activity and Function of the Nuclear Factor Kappa B Pathway. Oncogene.

[44] Tsai C.-Y., Li F.C.H., Wu C.H.Y., Chang A.Y.W., Chan S.H.H. (2016). Sumoylation of IkB Attenuates NF-KB-Induced Nitrosative Stress at Rostral Ventrolateral Medulla and Cardiovascular Depression in Experimental Brain Death. J. Biomed. Sci..

[45] Cánovas D., Marcos J.F., Marcos A.T., Strauss J. (2016). Nitric Oxide in Fungi: Is There NO Light at the End of the Tunnel?. Curr. Genet.

[46] Henard C.A., Vázquez-Torres A. (2011). Nitric Oxide and Salmonella Pathogenesis. Front. Microbio..

[47] Wiegand S.B., Traeger L., Nguyen H.K., Rouillard K.R., Fischbach A., Zadek F., Ichinose F., Schoenfisch M.H., Carroll R.W., Bloch D.B. (2021). Antimicrobial Effects of Nitric Oxide in Murine Models of Klebsiella Pneumonia. Redox Biol..

