# Supplementary material for: A Novel De Novo NFKBIA Missense Mutation Associated to Ectodermal Dysplasia with Dysgammaglobulinemia
Source: Genes (Basel). 2022 Oct 19;13(10):1900. doi: 10.3390/genes13101900 (PMC9602067; doi:10.3390/genes13101900)
Supplement: Supplementary file 1 [file genes-13-01900-s001.zip › genes-1944565-supplementary.pdf]

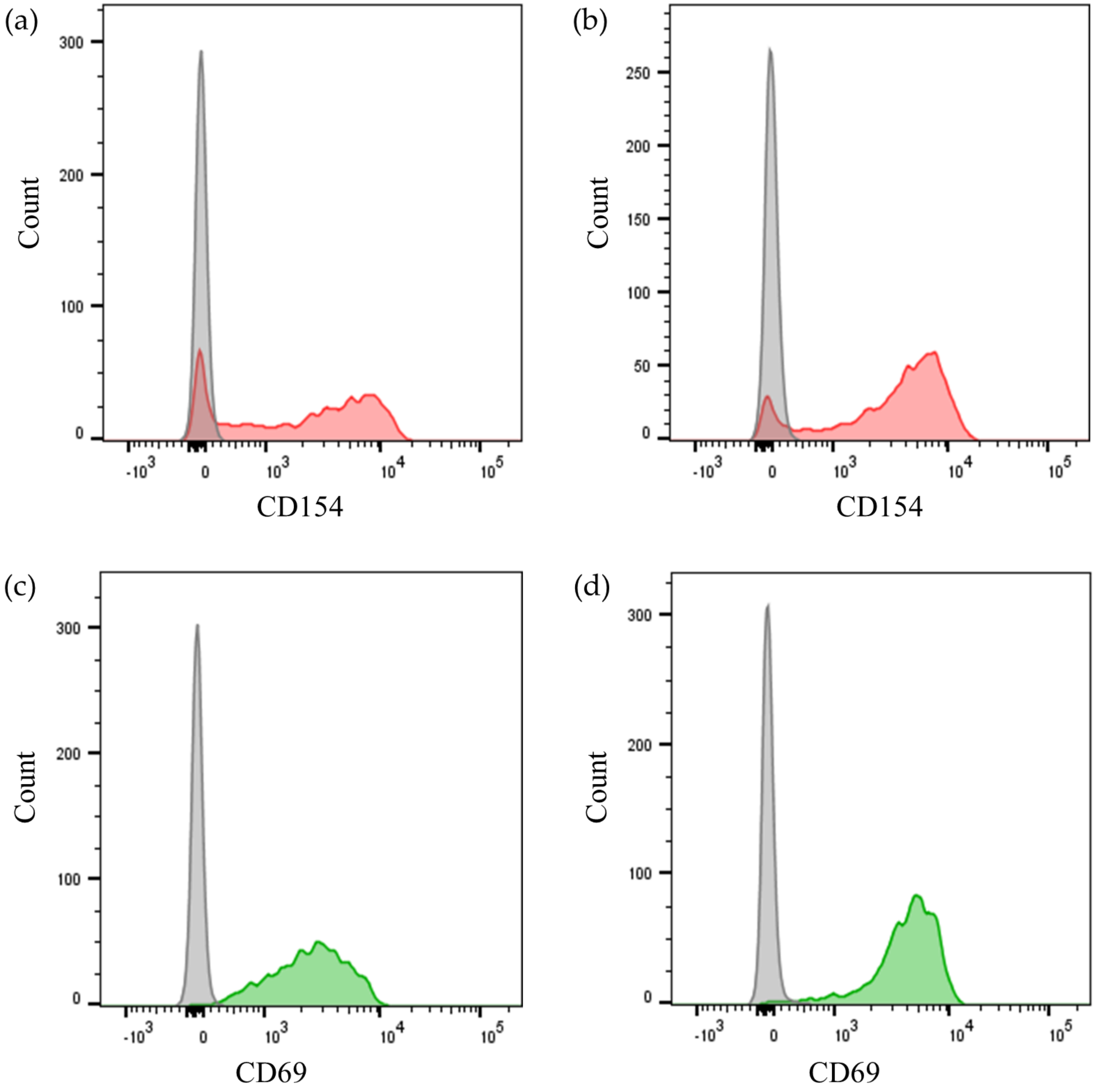

Figure S1. Cell surface expression of CD154 (CD40 ligand) on activated CD3+ CD4+ T-cells from healthy control and patient. Whole blood was stimulated *in vitro* with phorbol myristate acetate and calcium ionophore for 4 hours. The expression of CD154 and CD69 (a cell activation marker) were analyzed. The CD154 expression in stimulated cells (red histogram) is overlayed on unstimulated cells (grey histogram) of the (a) healthy control and (b) patient. The CD69 expression in stimulated cells (green histogram) is overlayed on unstimulated cells (grey histogram) of the (c) healthy control and (d) patient. Normal CD154 and CD69 expression are seen in the healthy control and patient.

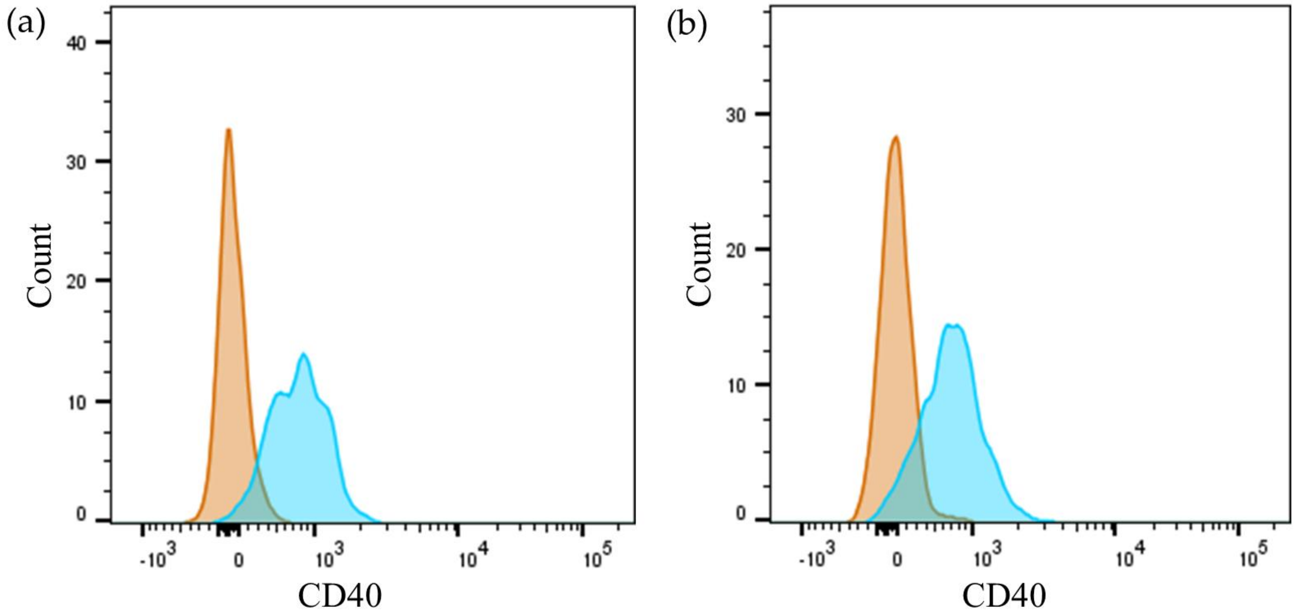

Figure S2. Flow cytometric analysis of CD40 expression in healthy control and patient. The CD40 expression on B cells (blue histogram) in the (a) healthy control and (b) patient were evaluated. The brown histogram represents the expression of isotype control. Both healthy control and patient had normal CD40 expression on B cells.

Table S1. Genes responsible for Ectodermal Dysplasias

**Gene symbol(s)**	**Gene full name**
ED-1	ectodysplasin A
EDAR	ectodysplasin A receptor
EDARADD	ectodysplasin A receptor associated death domain
NEMO/IKBKG	nuclear factor kappa B essential modulator/ inhibitor of nuclear factor kappa B kinase regulatory subunit γ
NFKBIA	nuclear factor kappa B inhibitor α
IKBKB	inhibitor of nuclear factor kappa B kinase subunit β
p63	tumor protein p63
DLX3	distal-less homeobox 3
MSX1	msh homeobox 1
EVC2	EvC ciliary complex subunit 2
EVC	EvC ciliary complex subunit 1
GJB6	gap junction protein beta 6
PVRL1	poliovirus receptor-like 1
PKP1	plakophilin 1
CDH3	cadherin 3
WNT10A	Wnt family member 10A

Table S2. List of five rare variants predicted to be damaging using *in silico* tools

**Gene**	**Mutation**	**MAF (gnomAD)**	**dbSNP**	**SIFT**	**Polyphen2**	**Mutation Taster**	**CADD phred**	**Genotype**	**ACMG classification**
SMARCAL1	*SMARCAL1*:NM_001127207: exon17:c.T2534A:p.L845Q	0.0000853	rs372298863	D	B	D	31	het	VUS with minor pathogenic evidence
TLR3	*TLR3*:NM_003265: exon4:c.C2384T:p.A795V	0.00005692	rs373118024	D	D	D	26.3	het	Uncertain significance
CHD7	*CHD7*:NM_017780: exon10:c.G2831A:p.R944H	0.0006	rs117506164	T	D	D	26.2	het	Benign
NFKBIA	*NFKBIA*:NM_020529: exon1:c.A94T:p.S32C	.	.	D	D	D	26.1	het	Pathogenic
ARHGEF1	*ARHGEF1*:NM_198977: exon5:c.G256A:p.A86T	0.000008132	rs371771035	D	D	N	23.5	het	Uncertain significance

B, benign; D, damaging; het, heterozygous; MAF, minor allele frequency; N, neutral; T, tolerated; VUS, variant with uncertain significance.

Table S3. List of variants in the genes related to ectodermal dysplasia

Gene	Mutation	MAF (gnomAD)	dbSNP	SIFT	Polyphen2	Mutation Taster	CADD phred	Genotype
PKP1	*PKP1*:NM_000299: exon3:c.A586G:p.I196V	0.0622	rs35507614	T	B	P	0.001	het
EDARADD	*EDARADD*:NM_145861: exon1:c.G27A:p.M9I	0.8412	rs966365	D	B	P	3.346	hom
EDARADD	*EDARADD*:NM_145861: exon1:c.G60A:p.E20E	0.0459	rs60808129	.	.	.	.	het
EDAR	*EDAR*:NM_022336: exon12:c.C1056T:p.C352C	0.8218	rs12623957	.	.	.	.	hom
EDAR	*EDAR*:NM_022336: exon9:c.C750T:p.S250S	0.9188	rs260632	.	.	.	.	hom
EVC2	*EVC2*:NM_001166136: exon20:c.C3267T:p.H1089H	0.3906	rs12511039	.	.	.	.	het
EVC2	*EVC2*:NM_001166136: exon14:c.A1855G:p.T619A	0.3639	rs730469	T	B	P	0.001	het
EVC2	*EVC2*:NM_001166136: exon5:c.A448G:p.S150G	0.2384	rs4689278	T	B	P	7.914	het
EVC	*EVC*:NM_001306090: exon6:c.C769T:p.L257L	0.9477	rs6446393	.	.	.	.	hom
EVC	*EVC*:NM_001306090: exon6:c.T772C:p.Y258H	0.7967	rs6414624	T	B	P	8.779	hom
EVC	*EVC*:NM_001306090: exon8:c.A1068G:p.L356L	0.2974	rs33929747	.	.	.	.	hom
EVC	*EVC*:NM_001306090: exon10:c.C1346A:p.T449K	0.7901	rs2302075	T	B	P	5.813	hom
EVC	*EVC*:NM_001306090: exon12:c.G1727A:p.R576Q	0.3486	rs1383180	T	D	P	24.9	het
EVC	*EVC*:NM_001306090: exon13:c.C1854T:p.G618G	0.3563	rs11737221	.	.	.	.	het
PVRL1	*PVRL1*:NM_203285: exon6:c.T1082G:p.V361G	0.9219	rs7940667	T	B	P	1.197	hom
NFKBIA	*NFKBIA*:NM_020529: exon2:c.C306T:p.A102A	0.1666	rs1050851	.	.	.	.	het
NFKBIA	*NFKBIA*:NM_020529: exon1:c.A94T:p.S32C	.	.	D	D	D	26.1	het
CDH3	*CDH3*:NM_001317195: exon16:c.A2334C:p.R778S	0.2614	rs3114409	T	.	P	0.085	het
DLX3	*DLX3*:NM_005220: exon2:c.G402A:p.T134T	0.2064	rs2303466	.	.	.	.	hom

B, benign; D, damaging; het, heterozygous; hom, homozygous; MAF, minor allele frequency; P, polymorphism; T, tolerated. The variant with MAF < 0.0001 is considered as rare variant. The pathogenic variant is highlighted in red.
